# DNA Clasping by Mycobacterial HU: The C-Terminal Region of HupB Mediates Increased Specificity of DNA Binding

**DOI:** 10.1371/journal.pone.0012551

**Published:** 2010-09-02

**Authors:** Sandeep Kumar, Abhijit A. Sardesai, Debashree Basu, Kalappagowda Muniyappa, Seyed E. Hasnain

**Affiliations:** 1 Laboratory of Molecular and Cellular Biology, Centre for DNA Fingerprinting and Diagnostics, Hyderabad, India; 2 Laboratory of Bacterial Genetics, Centre for DNA Fingerprinting and Diagnostics, Hyderabad, India; 3 Laboratory of Structural Biology, Centre for DNA Fingerprinting and Diagnostics, Hyderabad, India; 4 Department of Biochemistry, Indian Institute of Science, Bangalore, India; 5 Institute of Life Sciences, University of Hyderabad Campus, Hyderabad, India; 6 Department of Biochemistry, University of Hyderabad, Hyderabad, India; 7 Jawaharlal Nehru Centre for Advanced Scientific Research, Bangalore, India; East Carolina University School of Medicine, United States of America

## Abstract

**Background:**

HU a small, basic, histone like protein is a major component of the bacterial nucleoid. *E. coli* has two subunits of HU coded by *hupA* and *hupB* genes whereas *Mycobacterium tuberculosis* (*Mtb*) has only one subunit of HU coded by ORF *Rv2986c* (*hupB* gene). One noticeable feature regarding *Mtb* HupB, based on sequence alignment of HU orthologs from different bacteria, was that HupB*_Mtb_* bears at its C-terminal end, a highly basic extension and this prompted an examination of its role in *Mtb* HupB function.

**Methodology/Principal Findings:**

With this objective two clones of *Mtb* HupB were generated; one expressing full length HupB protein (HupB*_Mtb_*) and another which expresses only the N terminal region (first 95 amino acid) of hupB (HupB*_MtbN_*). Gel retardation assays revealed that HupB*_MtbN_* is almost like *E. coli* HU (heat stable nucleoid protein) in terms of its DNA binding, with a binding constant (K_d_) for linear dsDNA greater than 1000 nM, a value comparable to that obtained for the HUαα and HUαβ forms. However CTR (C-terminal Region) of HupB*_Mtb_* imparts greater specificity in DNA binding. HupB*_Mtb_* protein binds more strongly to supercoiled plasmid DNA than to linear DNA, also this binding is very stable as it provides DNase I protection even up to 5 minutes. Similar results were obtained when the abilities of both proteins to mediate protection against DNA strand cleavage by hydroxyl radicals generated by the Fenton's reaction, were compared. It was also observed that both the proteins have DNA binding preference for A:T rich DNA which may occur at the regulatory regions of ORFs and the *oriC* region of *Mtb*.

**Conclusions/Significance:**

These data thus point that HupB*_Mtb_* may participate in chromosome organization *in-vivo*, it may also play a passive, possibly an architectural role.

## Introduction

The *Escherichia coli* (*E. coli*) protein HU (heat stable nucleoid protein) is an abundant DNA binding protein, which is a major component of the bacterial nucleoid [1]. HU is a small, basic, histone like protein, initially called factor U, isolated for the first time from *E. coli* strain U93 [1], [2]. In *E. coli* HU activity is comprised of a hetero-dimer of HupA (Hu α) and HupB (Hu β) coded by *hupA* and *hupB* genes, respectively. Besides this, homo-dimeric forms (Hu α2 and Hu β2) are also observed with different forms (mainly Hu α2 and Hu αβ) predominating in distinct phases of growth [3]. HU is highly conserved [4] and unlike most DNA binding proteins, binds to both single stranded (ss) and double stranded (ds) DNA in a sequence independent manner [5], [6]. The other major nucleoid associated proteins are FIS (factor for inversion stimulation), H-NS (histone like nucleoid protein), IHF (integration host factor) and a stationary phase specific DNA binding protein, DPS (DNA binding protein from starved cells [2], [7], [8]. HU, which resembles eukaryotic proteins of the high mobility group (HMG) class in terms of its DNA binding properties e.g. it binds dsDNA with low affinity and negligible sequence specificity; also displays high affinity for some altered DNA structures such as junctions, nicks, gaps, forks, and overhangs even under stringent salt conditions [9], [10]. In *E. coli* HU action is fairly pleiotropic in the sense that HU plays a role in many cellular processes such as site specific recombination [11], [12], initiation of DNA replication [13], [14], phage Mu transposition [15] and introducing negative supercoiling into relaxed DNA molecule in presence of Topoisomerase I [2]. Furthermore, cells deficient for HU are known to be highly sensitive to gamma and UV irradiation and it is thought that HU might assist in the processes of recombinational repair [11], [12].

HU also mediates ring closure of linear DNA enhancing DNA cyclization rates [16]. Crystal structure of Anabaena HU bound to DNA revealed that binding of dimers of HU to linear DNA with cohesive ends produces an overall bend of ∼105–140° thus stimulating rate of ring formation [17]. Once cohesive DNA ends are brought together they can be sealed by T_4_ DNA ligase [16], [18], [19]. *E. coli* HU binds to linear DNA fragments in a weakly cooperative fashion with one dimer occupancy per 9 bp and this binding is observed only under low salt conditions [20]. The binding of HU to a DNA four way junction is several orders of magnitude higher in comparison to binding to linear DNA even under high salt conditions [21] and is not inhibited by a large (100 fold) excess of competitor linear DNA. This binding preference is thought to be commensurate with the role of HU in DNA inversion [22].

Although in *E. coli* the heterodimeric state of HU is quite preponderant, in many bacterial species HU exists as a homodimer like in *Bacillus subtilis* [23], [24], Mycobacterial family etc. The annotated genome of *Mycobacterium tuberculosis* strain H37Rv, (*Mtb*) bears the potential to encode only one subunit of HU - the product of ORF *Rv2986c* (*hupB_Mtb_*). The product of ORF *Rv2986c* is also referred to as mycobacterial DNA binding protein (MDP-1) or histone like protein (HLP_Mt_) [25], [26]. HupB*_Mtb_* is 214 amino acids long, has a high content of alanine (23.78%) and lysine (18.93%) and has a calculated pI of 12.4 with the ratio of basic to acidic amino acid residues being 12. This ratio in HupA and HupB of *E. coli* is 1.4, while for histone H1 or H5 this ratio is approximately 7 [25]. The N-terminal portion of HupB*_Mtb_* exhibits significant homology to histone like proteins of *E. coli* while the C-terminal part displays homology to eukaryotic H1 histone [25]. Sequence alignment of HU homolog's from different members of the *Mtb* complex shows that N terminal end of HupB is conserved, but the C-terminal end is variable (Supplementary Figure S2) and this feature has therefore been used as a diagnostic marker for differentiating members of *Mtb* complex [27]. HupB*_Mtb_*, like other bacterial HU proteins, lacks tryptophan, cysteine and tyrosine residues. These properties and an overall homology to bacterial HU suggest that HupB*_Mtb_* could be involved in the packaging of mycobacterial DNA and functions as a nucleoid associated protein. Interestingly HupB*_Mtb_* interacts with the immune system, an interaction that may occur due to release of protein during natural cell lysis or release resulting from interaction with the host immune system [25], [28]. Recently HupB*_Mtb_* ortholog in *M. smegmatis* has been shown to possess DNA end joining/ring closure protein activity [19].

In this report we describe biochemical studies on recombinant, purified HupB*_Mtb_* and show that it displays properties of a nucleoid associated protein in terms of non-specific DNA binding activity. By undertaking comparative studies on HupB*_Mtb_* and a derivative that lacks 119 amino acids located at the C-terminal end, we provide evidence to show that this C-terminal region (CTR) of HupB*_Mtb_* is required for providing increased specificity of DNA binding and for specific recognition of altered nucleic acid structures under stringent (high salt) conditions. DNA binding by HupB*_Mtb_* is stable and, in contrast to its derivative lacking the CTR, protects DNA from attack by reactive oxygen species produced by the Fenton's reagent and also affords substantial protection against cleavage by DNaseI. HupB*_Mtb_* displayed substantial preference in binding to AT rich DNA. These observations suggest that HupB*_Mtb_* action is different from enterobacterial HU and the CTR of HupB*_Mtb_* may act like a DNA clasp. Given its increased preference to bind to AT rich DNA it is speculated that the site(s) of HupB*_Mtb_* action on the *Mtb* genome could be AT rich regions that are found in the replication origin and regulatory regions of genes.

## Results

### Presence of a basic CTR in HupB orthologs from the mycobacterial clade

As noted previously a very visible feature of the polypeptide sequence of HupB orthologs belonging to the mycobacterial clade is the presence of a basic CTR [25], [27]. Member proteins of this clade are roughly 214 amino acids long whereas *E. coli* HupA and HupB are 90 amino acids long. The N terminal region of HupB*_Mtb_* shows significant homology to individual subunits of enterobacterial HU namely HupA, HupB from *E. coli*, HupB from *S. enterica* and HU from *B. subtilis* (Supplementary Figure S1A), whereas the CTR despite retaining its overall basic character displays some variation in amino acid composition within members of the mycobacterial clade (Supplementary Figure S2). Amino acid sequence of the HupB*_Mtb_* CTR shows that it is rich in lysine and alanine. It has six PAKK and one KAAK repeats (Supplementary Figure S1A and Supplementary S1B,) which are also present in histone H1, and are known to facilitate DNA binding [29]. The CTR of HupB*_Mtb_* also shows significant homology to sea urchin histone H1 (Supplementary Figure S1B) primarily in terms of the presence of these tetrapeptide repeats. In order to determine the specific function related to this CTR, two *hupB_Mtb_* ORFs were generated by PCR; one bearing the full length *hupB_Mtb_* DNA sequence (encoding HupB*_Mtb_*) and another encoding a *hupB_Mtb_* variant bearing only the N-terminal 95 amino acids (HupB*_MtbN_*).

### The CTR of HupB*_Mtb_* imparts increased specificity in DNA binding

**Figure 1:**
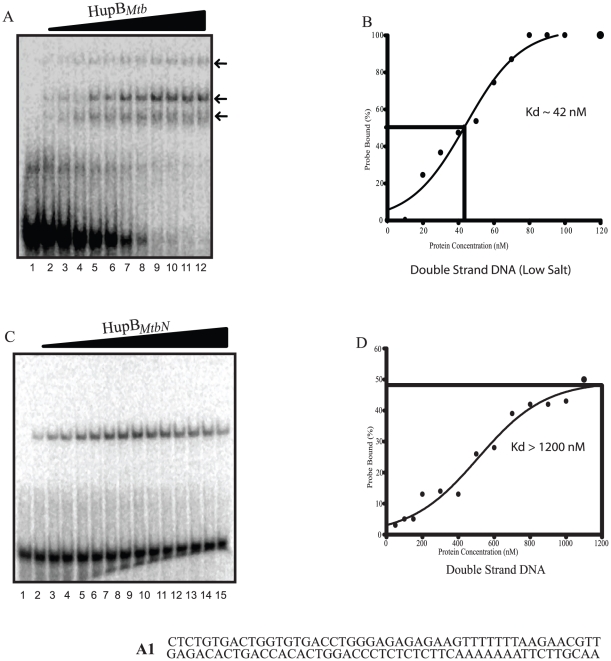
HupB*_Mtb_* shows higher binding affinity to double stranded DNA. Comparative gel retardation analysis showing the binding of increasing amounts of (A) HupB*_Mtb_* (0–120 nM, lanes 1–12) and (C) HupB*_MtbN_* (0–1200 nM, lanes 1–15) to radioactively labeled ds DNA under low salt conditions (10 mM KCl) (sequences shown in Table 2). Dissociation constants (Kd) were also calculated (B) for HupB*_Mtb_* and (D) for HupB*_MtbN_* binding to ds DNA. The Kd values are indicated as inset in the respective panels. Sequence of the oligonucleotide A1 used here is provided at the bottom of the figure.

**Figure 2:**
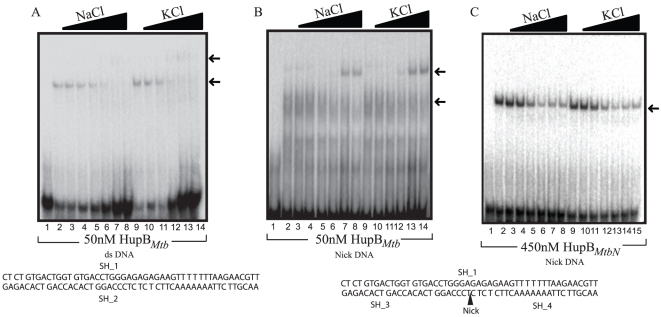
HupB*_Mtb_* binds specifically to nick DNA under high salt conditions. Comparative gel retardation analysis showing the binding of (A) 50 nM of HupB*_Mtb_* under increasing salt concentration (0, 10, 50, 100, 150, 200 and 250 mM) of either NaCl (lanes 1–7) or KCl (lanes 8–14) as indicated in figure, to DNA (Table 2) ds or (B) nick DNA. (C) 450 nM of HupB*_MtbN_* binding to nick DNA under increasing salt concentration (0, 10, 50, 100, 150, 200 and 250 mM) of either NaCl or KCl as indicated in figure. Position of the nick in the DNA is marked by an arrowhead.

Under low salt conditions (10 mM NaCl or KCl) HupB*_Mtb_* bound to linear ds DNA (ds oligonucleotide A1) in a weakly cooperative manner with a Kd of 42 nM whereas even at 1200 nM HupB*_MtbN_* barely displayed 50% binding to A1. (Figure 1A and 1B). For HupB*_Mtb_* linear ds DNA interaction three retarded complexes (Figure 1A shown by arrowheads) were apparent with complex 2 being the major species. However with HupB*_MtbN_* only a single band could be observed (Figure 1C) which shows that the binding is non-cooperative. Based upon the length of ds DNA used herein (that is 48 bp) and assuming that retardation is provoked by dimeric HupB*_Mtb_*, one can roughly estimate that HupB*_Mtb_* binds to linear DNA with one dimer occupying DNA ranging from 16 to 24 bp. Binding of HupB*_Mtb_* (at 50 nM) to A1 under increasing salt concentrations revealed that HupB*_Mtb_* binding was greatly impaired at salt concentrations above 150 mM (NaCl or KCl; Figure 2A). *E. coli* HU is known to bind with duplex DNA containing a nick or a gap of one or two nucleotides with high affinity [30]. A specific DNA protein complex (Kd 65 nM), under high salt conditions was detected when the interaction of HupB*_Mtb_* with linear DNA bearing a nick (ds oligonucleotide A2) was studied (Figure 2B). On the other hand, binding of higher concentrations of HupB*_MtbN_* (employed at 450 nM) promoted binding to A2 under low salt conditions, which was impeded under high salt conditions (Figure 2C). Under low salt HupB*_MtbN_* bound to nicked DNA with a Kd of 1000 nM (Table 1). HU is known to bind specifically to DNA containing either nick or a gap or DNA junction (cruciform DNA) without sequence preference [9], [10], [30]. These structures are associated with DNA damage and repair.

**Table 1:** Binding constants (Kd) of HupB*_Mtb_* and HupB*_MtbN_* to different structures of nucleic acid.

DNA Structure	HupB*_Mtb_*		HupB*_MtbN_*
	Kd (200 mM KCl)	Kd (10 mM KCl)	Kd (10 mM KCl)
**ds DNA**	-	42 nM	>1200 nM
**Nick DNA**	65 nM	-	1000 nM
**1 nucleotide gap DNA**	-	55 nM	725 nM
**2 nucleotide gap DNA**	88 nM	60 nM	536 nM
**Cruciform DNA**	66 nM	60 nM	160 nM

**Figure 3:**
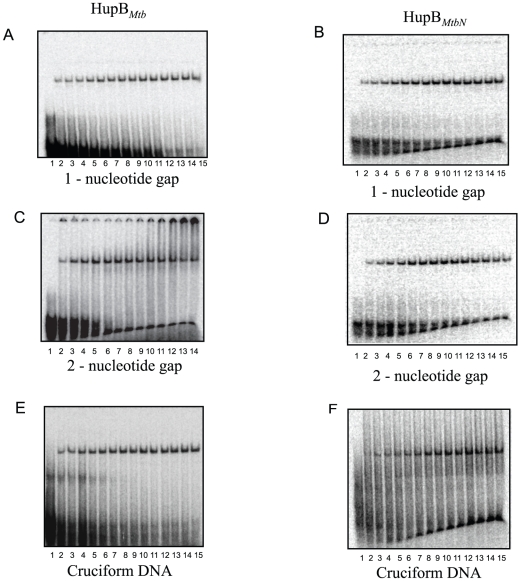
HupB*_Mtb_* binds with high affinity to 1-nucleotide gap, 2-nucleotide gap and cruciform DNA under low salt (10 mM KCl) in comparison to HupB*_MtbN_*. Gel retardation analysis showing the binding of increasing amounts of (A) HupB*_Mtb_* (0–210 nM) and (B) HupB*_MtbN_* (0–1200 nM) to 1-nucleotide gap DNA (C) HupB*_Mtb_* (0–210 nM) and (D) HupB*_MtbN_* (0–1200 nM) to 2-nucleotide gap DNA (E) HupB*_Mtb_* (0–210 nM) and (F) HupB*_MtbN_* (0–1200 nM) to cruciform DNA. The gel retardation conditions are described in Materials and Methods.

In order to address the question whether HupB*_Mtb_* and HupB*_MtbN_* displayed differential binding we studied their interaction with linear ds DNA bearing one or two nucleotide gap and cruciform DNA, represented by ds oligonucleotides A3, A4 and A5 respectively, in the presence of low salt (10 mM KCl) (Supplementary Figure S3 and Supplementary Figure S4; for details refer Supplementary Methods S1). It is apparent (Figure 3; Table 1) that the CTR of HupB*_Mtb_* is required for specific recognition and high affinity recognition of a duplex DNA containing a nick or a gap of one or two nucleotides and cruciform DNA.

### Interaction of HupB*_Mtb_* with supercoiled plasmid DNA

**Figure 4:**
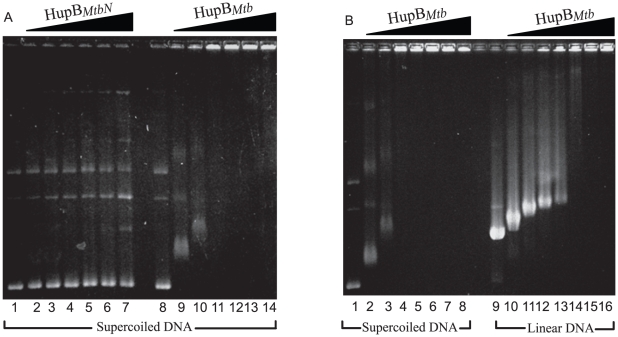
HupB*_Mtb_* binds strongly to supercoiled and linear plasmid DNA in comparison to HupB*_MtbN_* as evident from agarose gel electrophoresis of pBluescript SK (linear and supercoiled). **A**) 500 ng of supercoiled pBluescript SK plasmid was incubated with increasing amounts (0, 1.2, 0.5, 1.0, 1.5, 2.0 and 2.5 µM) of either HupB*_MtbN_* (lanes 1–7) or HupB*_Mtb_* (lanes 8–14) in low salt conditions (10 mM KCl). The DNA-protein interaction products were resolved by electrophoresis on a 1% agarose gel buffered with 0.5× TBE at 35 V, 16–20 hrs. **B**) 500 ng of either supercoiled (lanes 1–8) or linear (lanes 9–16) pBluescript SK DNA was incubated with increasing amount of HupB*_Mtb_*. The DNA-protein interaction products were resolved as described above.

Despite a lack of sequence specificity in DNA binding, HU is known to bind preferentially to negatively supercoiled DNA [17], [31]. The interaction of HupB*_Mtb_* and HupB*_MtbN_* with supercoiled pBluescriptSK (pBSK) plasmid DNA was studied in buffer conditions such that the molar ratio of protein to DNA ranged from 15∶1 to 180∶1. It could be seen that HupB*_MtbN_* displayed a markedly reduced interaction with supercoiled plasmid DNA (Figure 4A, lanes 1–7). Even at the highest protein to DNA molar ratio sufficient amount of unbound pBSK plasmid was visible (Figure 4A, lane 7). HupB*_Mtb_* on the other hand bound very proficiently with supercoiled DNA, in the sense that at intermediate protein to DNA molar ratio the intensity of DNA protein complex was large and the DNA remained lodged in the wells Figure 4A, lanes 11 to 14). This property is reminiscent of proteins that can induce DNA compaction [32]. In order to test the preference of HupB*_Mtb_* towards supercoiled/linear DNA, pBluescript SK (pBSK) plasmid was linearized using *Eco*R1. EMSA was carried out with supercoiled pBSK (Figure 4B, lanes 1–8) and with linear pBSK (lanes 9–16). It was observed that interaction of HupB*_Mtb_* with supercoiled pBSK at a molar ratio (protein: DNA) of 75∶1 the intensity of DNA protein complex was large and the DNA remained lodged in the well (lane 4). However, the interaction of HupB*_Mtb_* with linear pBSK was observed at a molar ratio of 112.5∶1 (lane 14). It therefore appears that HupB*_Mtb_* exhibits ∼1.5-fold higher affinity towards supercoiled plasmid DNA. Furthermore, the interaction of HupB*_Mtb_* with supercoiled DNA was more avid than with linear DNA of the same size (Figure 4B lanes 9–16).

### HupB*_Mtb_* displays greater affinity in binding to AT rich DNA

**Figure 5:**
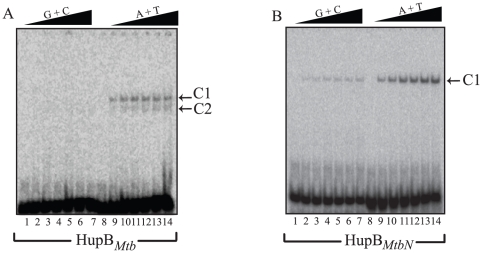
HupB*_Mtb_* displays higher affinity for AT rich DNA. Electrophoretic mobility shift assay was carried out with GC rich ds DNA (A6) or AT rich ds DNA (A7) with increasing amount of either (A) HupB*_Mtb_* (0–300 nM) or (B) HupB*_MtbN_* (0–300 nM). Lanes 1–7 of both the panels have GC rich oligo with protein concentrations ranging from 0, 50, 100, 150, 200, 250 and 300 nM. Similarly, lanes 8–14 of both the panels have AT rich oligo with protein concentrations ranging from 0, 50, 100, 150, 200, 250 and 300 nM, respectively.

Since the average GC content of the *Mtb* H37Rv genome works out to around 65%, we wondered whether the CTR of HupB*_Mtb_* would endow it with the ability to interact more proficiently with GC rather than AT rich DNA. To assess whether HupB*_Mtb_* bore this attribute, interaction of this protein was studied with ds DNA oligonucleotides A6, (GC content 84%) and A7 (AT content 64%). Surprisingly, HupB*_Mtb_* interacted proficiently with AT rich DNA forming two gel-retarded complexes (C1, C2; Figure 5A marked by arrowheads) where as its interaction with GC rich DNA was negligible. On the other hand HupB*_MtbN_* while retaining its ability to interact more proficiently with AT rich DNA, forming a single gel retarded complex (Figure 5B, lanes 9–14), displayed weak interaction with GC rich DNA (Figure 5B, lanes 2–7). We then determined by circular dichroism spectrometry the secondary structure parameters of the two proteins, which revealed that the two proteins exhibited some differences in the content of alpha helix and beta sheet structures (Supplementary Figure S5; for details refer Supplementary Methods S1). Perhaps these differences may account for the ability of HupB*_MtbN_* to display binding to GC rich DNA.

### Enhanced DNA protection mediated by the CTR of HupB*_Mtb_*

**Figure 6:**
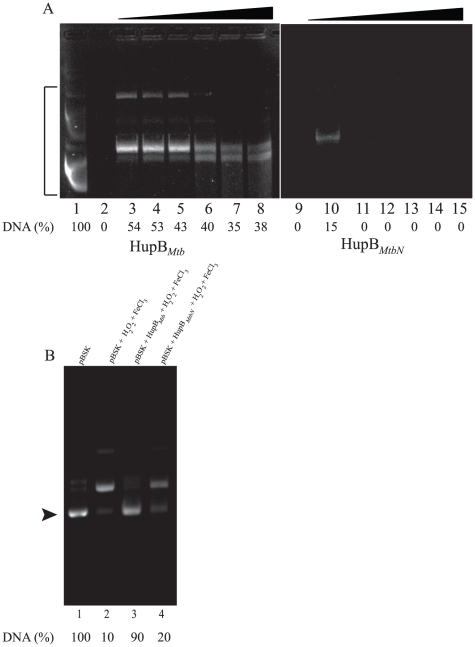
CTR of HupB*_Mtb_* provides protection from DNaseI. (**A**) Timed digestion of supercoiled pBSK DNA with DNase I was carried out with 2.5 µM of HupB*_Mtb_* (A, lanes 1–8) or HupB*_MtbN_* (lanes 9–15). Lanes 1, 2 and 9 have no protein while lanes 3–8 and lanes 10–15 have 2.5 µM of HupB*_Mtb_* and HupB*_MtbN_*, respectively. The reaction was initiated by the addition of one unit of DNaseI to all the lanes except lane 1. Incubation time with DNaseI in lanes 2–3 and 9–10 was 30 sec; lanes 4 and 11 for 1 min; lanes 5 and 12 for 2 min; lanes 6 and 13 for 3 min, lanes 7 and 14 for 4 min and lanes 8 and 15 for 5 min respectively. Area quantitated in this figure is marked by bracket and the amount of DNA (%) in each lane is indicated at the bottom of figure. (**B**) Protection of DNA from H_2_O_2_ mediated damage. Lane 1, pBSK DNA alone; lane 2, pBSK treated with FeCl_3_ and H_2_O_2_; lane 3, first incubated with HupB*_Mtb_* and then treated with FeCl_3_ and H_2_O_2_ and lane 4, first incubated with HupB*_MtbN_* and then treated with FeCl_3_ and H_2_O_2_ as indicated in material and methods. The lower band in Figure 6B represents supercoiled DNA while the upper band represents relaxed DNA. The band marked by arrowhead was quantitated and the amount of DNA (%) in each lane is indicated at the bottom of figure.

Given that the CTR of HupB*_Mtb_* imparts upon it increased avidity with respect to DNA binding we tested whether the said region could protect DNA from enzymatic or non-enzymatic DNA strand breakage. In the first instance we tested the abilities of HupB*_Mtb_* and HupB*_MtbN_* to mediate protection of DNA from cleavage by DNaseI. Supercoiled pBSK plasmid was exposed to 1 unit of DNase I for 30 seconds at 25°C in the presence of varying concentrations of HupB*_Mtb_* and HupB*_MtbN_* (0.2–2.5 µM). Furthermore, timed digestions with DNase I were carried out with 2.5 µM of HupB*_Mtb_* and HupB*_MtbN_* (Figure 6A and 6B). While both proteins at 2.5 µM gave substantial protection to DNA from DNase I cleavage (data not shown), analysis of timed digestion with DNaseI revealed that the binding of HupB*_Mtb_* was stable enough to provide protection from DNase I even up to 5 min (Figure 6A, lane 8). However HupB*_MtbN_* was significantly impaired in mediating protection from DNase I, and could not provide protection beyond 30 sec (Figure 6A, lanes 11–15), as could be seen from the absence of DNA band (lanes 11–15). Similar results were obtained when the abilities of both proteins to mediate protection against DNA strand cleavage by hydroxyl radicals generated by the Fenton's reaction, were compared. HupB*_Mtb_* was significantly more proficient than HupB_MtbN_ in preventing DNA strand cleavage upon exposure to the Fenton system (Figure 6B). These studies thus suggest that the CTR of HupB*_Mtb_* promotes associations with DNA that are stable enough to promote substantial DNA protection.

## Discussion

In bacteria the chromosomal DNA is present in a highly compacted form, called the bacterial nucleoid, in which DNA exists in association with basic low molecular weight proteins. Unlike that seen for eukaryotic chromatin, bacterial chromosome lacks perceptible DNA protein organization. In the well-studied case of *E. coli*, low molecular weight proteins such as H-NS, HU, IHF and FIS are thought to be the key protein constituents of the bacterial nucleoid [7], [8], [33], [34]. While both HU and H-NS are very abundant, HU has been considered to be a prokaryotic histone, primarily based upon its resemblance to eukaryotic histones in terms of its non-specific DNA binding capacity and amino acid sequence composition [2], [8]. HU is highly conserved and in enterobacteriacae HU exists as a heterodimer comprising of two homologous protein subunits, Huα and Huβ. The heterodimeric state of HU appears to be restricted to enterobacteriacae, whereas in many other bacteria HU exists as a homodimer.

In this study we have examined the biochemical properties of the HU ortholog from *M. tuberculosis* (*Mtb*) strain H37Rv, the product of ORF *Rv2986c*, designated as HupB*_Mtb_*. One noticeable feature regarding HupB*_Mtb_*, based upon sequence alignment of HU orthologs from different bacteria, was the presence at its C-terminal end a highly basic extension (Supplementary Figure S1), designated as the C-terminal region (CTR), which prompted us to examine its role. Using purified recombinant HupB*_Mtb_* and HupB*_MtbN_*, a variant of the former that lacks the CTR, comparative biochemical studies were undertaken. HupB*_Mtb_* and HupB*_MtbN_* exhibited HU-like DNA binding properties, in that both displayed non-specific DNA binding with the difference that HupB*_Mtb_* was more proficient in binding to DNA than HupB*_MtbN_* in several DNA binding assays employed. These results therefore suggest that the CTR of HupB*_Mtb_* imparts upon it greater specificity in DNA binding (Figure 1 and Figure 2). Recently, HU ortholog from *M.smegmatis* has been shown to possess similar properties [19]. It is worth noting that HupB*_MtbN_* is almost *E. coli* HU- like in terms of its DNA binding, its DNA binding constant (K_d_) with linear dsDNA is greater than 1000 nM, a value comparable to that obtained for the HUαα and HUαβ forms [10]. Furthermore, HupB*_MtbN_* like *E. coli* HU displayed comparatively greater affinity towards DNA bearing a nick or gap and cruciform DNA (Supplementary Figure S4, Table 1). In all these instances DNA binding affinities of HupB*_Mtb_* were comparatively greater. These observations suggest that the basic or skeletal DNA binding property of HupB*_Mtb_* resides in its first 90 amino acid residues and that the basic CTR acts to enhance DNA binding.

Recent crystal structure studies of Anabaena HU (homodimeric protein of size similar to and homologous to *E. coli* HU) have shown that in the HU dimer, two monomers associate to form an α-helical base extending outwards from each monomer and contribution from its C-terminal region is a largely β-sheeted structure (extends as two β-ribbon arms that contact the DNA within its minor groove) [17], [35]. The N-terminal half of each monomer contributes the α-helical base. The presence of a highly conserved proline residue at position 63 (present in HupB*_Mtb_* and HupB*_MtbN_*, Supplementary Figure S1) at the tip of each arm is thought to be critical for DNA binding. Given these observations, it is possible to place the presence of the basic DNA binding property of HupB*_Mtb_* in its N-terminal 90 amino acids. HupB*_Mtb_* displayed more stable DNA associations than HupN*_MtbN_*, HupB*_Mtb_* bound more proficiently to supercoiled plasmid DNA and, commensurate with this property, mediated enhanced protection of DNA from enzymatic or non-enzymatic DNA strand breakage (Figure 4). From these studies it appears that the CTR of HupB*_Mtb_* may act like a DNA clasp promoting more stable DNA interactions. It is apparent that though presence of CTR lowers the K_d_ of dsDNA binding by at least one order of magnitude in comparison to HupB*_MtbN_*, (and *E. coli* HU), the K_d_ (42 nM for HupB*_Mtb_*) is not lowered to the extent expected for a site specific DNA binding protein. Thus, HupB*_Mtb_* bears the propensity to function as a nucleoid-associated protein in its natural setting.

An interesting finding was that HupB*_Mtb_* interacted much more proficiently with substantially A:T rich than with G:C rich DNA. The G:C content of the *Mtb* genome is around 65% and is fairly uniform. Admittedly the G:C content of the dsDNA oligonucleotide A13 used in these studies is high (84%), nevertheless it is present in the *Mtb* genome located upstream of the ORF *Rv494*. On the other hand HupB*_Mtb_* bound proficiently with dsDNA oligonucleotide A1 that constitutes a DNA sequence located within the origin of chromosomal replication on the *Mtb* genome, present between the *dnaA* and the *dnaN* genes. Recently we showed that *Mtb* DnaA *in-vitro* can mediate DNA duplex unwinding in the vicinity of this sequence [36]. At the *E. coli* origin of replication *oriC*, strand opening by DnaA is known to require the presence of either HU or IHF [14]. It is thus possible that the site(s) of biological HupB*_Mtb_* activity in *Mtb* may be restricted to A:T rich regions, at least one that is present in the origin of replication where HupB*_Mtb_* could promote efficient DnaA mediated helix unwinding and/or to other regions of limited A:T richness that may occur at the regulatory regions of ORFs. It is interesting to note that HupB*_Mtb_* is considered to be essential for mycobacterial growth, an observation that is apparently compatible with its suspected role in DNA replication [37]. Future studies in this regard, directed towards exploring the possible requirement of HupB*_Mtb_* in the initiation of chromosomal DNA replication are underway.

## Materials and Methods

### Cloning and purification of recombinant His tagged HupB*_Mtb_* and HupB*_MtbN_* protein

The *M. tuberculosis* ORF *Rv2986c* coding for HupB*_Mtb_* and HupB*_MtbN_* protein was PCR amplified using genomic DNA from H37Rv as a template and primers HupB*_Mtb_* _F, HupB*_Mtb_* _R and HupB*_MtbN_*_R, carrying specific restriction enzyme sites (Table 2), by Accutaq DNA polymerase (Sigma). The amplicons thus generated were digested with *Nde*1/*Hin*dIII restriction enzymes and cloned into the corresponding sites of pET28a expression vector. The resultant plasmids were labeled as pETHupB*_Mtb_* and pETHupB*_MtbN_*. Restriction analysis and DNA sequencing confirmed the authenticity of all constructs. Recombinant HupB*_Mtb_* and HupB*_MtbN_*, coded by *M.tb* ORF *Rv2986c*, were purified from the soluble fraction of BL21 (DE3) cells transformed with pETHupB*_Mtb_* and pETHupB*_MtbN_*. The transformants were grown overnight at 18°C and induced with 0.15 mM IPTG at an OD_600_ of 0.2 for the expression of recombinant protein as described earlier [38], [39]. The recombinant protein was purified in buffer having 30 mM Tris (pH 8.5), 5 mM MgCl_2_, 250 mM NaCl, 100 mM potassium glutamate and 7% glycerol. SDS PAGE confirmed the purity of the protein. The concentration of the protein was estimated by BCA (Bichinconic acid) and protein was stored at −20°C until further use.

**Table 2:** Sequence of oligonucleotides used for Electrophoretic Mobility Shift Assays.

Oligonucleotide name	Oligonucleotide sequence	Oligonucleotide length
HupB*_Mtb_* _F	GGAATTCATATGAACAAAGCAGAGCTCATTGACG	34
HupB*_Mtb_* _R	GCAAGCTTCTATTTGCGACCCCGCCGA	27
HupB*_MtbN_*_R	GCAAGCTTCTAGAGACGCTGCGCGCCAGAC	31
SH_1	CTCTGTGACTGGTGTGACCTGGGAGAGAGAAGTTTTTTTAAGAACGTT	48
SH_2	AACGTTCTTAAAAAAACTTCTCTCTCCCAGGTCACACCAGTCACAGAG	48
SH_3	GAGACACTGACCACACTGGACCCT	24
SH_4	CTCTCTTCAAAAAAATTCTTGCAA	24
SH_5	TCTCTTCAAAAAAATTCTTGCAA	23
SH_6	CTCTTCAAAAAAATTCTTGCAA	22
SH_7	GAACTGACCGGACTGGACGAGCGCGAGAGAAGTTTTTTTAAGAACGTT	48
SH_8	AGGACGGCAATTACTCGCCGCAGCGCGCTCGTCCAGTCCGGTCAGTTC	48
SH_9	CTCTGTGACTGGTGTGACCTGGGAGCTGCGGCGAGTAATTGCCGTCCT	48
SH_10	GCCCGGCTGCACCGCGCCACCGCGG	25
SH_11	CCGCGGTGGCGCGGTGCAGCCGGGC	25
SH_12	TCAAATCTAATCGGAGTCGTTTTGA	25
SH_13	TCAAAACGACTCCGATTAGATTTGA	25

ds DNA formed after annealing	Oligonucleotide used for annealing	
A1 (ds DNA)	SH_1 + SH_2	
A2 (nick DNA)	SH_1 + SH_3 + SH_4	
A3 (1 nt. gap DNA)	SH_1 + SH_3 + SH_5	
A4 (2 nt. gap DNA)	SH_1 + SH_3 + SH_6	
A5 (Cruciform DNA)	SH_1 + SH_7 + SH_8 + SH_9	
A6 (84% GC DNA)	SH_10 + SH_11	
A7(64% AT DNA)	SH_12 + SH_13	

### Electrophoretic mobility shift assays

The interaction of increasing amounts of protein (0.2–2.5 µM) (HupB*_Mtb_* and HupB*_MtbN_*) with 480 ng of supercoiled or linearized plasmid DNA (pBSK linearized with *Eco*R1) was carried out in buffer A (20 mM HEPES-KOH pH 7.5, 5 mM magnesium acetate, 1 mM EDTA, 0.05 mM bovine serum albumin and 7% glycerol). The reactions were incubated at 25°C for 30 min and the products resolved by electrophoresis on a 1% agarose gel in 0.5× TBE at 35 V for 16–20 hrs. The gels were visualized by staining with ethidium bromide.

The interaction of HU (HupB*_Mtb_* and HupB*_MtbN_*) protein with dsDNA oligonucleotide probes was characterized by native PAGE. Increasing amounts of protein was incubated with 5′^32^P-labelled DNA for 30 min at room temperature in 20 µl of binding buffer A. The DNA protein complex were resolved on a 5% polyacrylamide gel (29∶1) buffered with 0.25× TBE as mentioned in figure legends. The binding of HU protein (HupB*_Mtb_* and HupB*_MtbN_*) to nicked, 1-nt gap, 2-nt gap or cruciform DNA was carried out in buffer A, either in low salt (10 mM KCl) or in high salt (200 mM KCl) conditions. Any variations apart from the above-mentioned conditions are specified in the figure legends.

### DNaseI protection Assay

The DNaseI protection assays were carried out in presence of 500 ng of pBluescriptK plasmid either in presence of increasing protein concentrations (HupB full length and mutant protein) (0.2–2.5 µM) or constant protein concentration (2.5 µM). The reactions were performed in buffer B (20 mM HEPES-KOH pH 7.5, 5 mM magnesium chloride, 1 mM EDTA, 0.05 mM bovine serum albumin and 7% glycerol) and were incubated for 30 min at 25°C. The DNaseI digestion was initiated by the addition of 1 unit of DNaseI (PROMEGA, USA) for 30 sec or an increasing time interval ranging from 30 sec to 5 minutes as mentioned in figure legends. The reactions were stopped by the addition of 3 µl of 20% SDS and 2 µl of 0.5 M EDTA. The reaction products were subsequently resolved by electrophoresis on 1% agarose gel in 1× TAE.

### DNA protection from metal catalyzed oxidation system

The ability of HupB*_Mtb_* and HupB*_MtbN_* to protect 500 ng of plasmid pBluescript II SK (+) from Metal catalyzed oxidation system (MCO) was tested in presence of either 1 µM of HupB*_Mtb_* or HupB*_MtbN_* in a reaction volume of 15 µl for 30 minutes at room temperature. MCO (0.4 µM FeCl_3_, 10 mM DTT, 100 mM Ethanol and 2 mM H_2_O_2_) was added to the protein DNA complex and the reaction was further continued for 30 min. The reaction was terminated by the addition of 10 mM EDTA, phenol extracted and analyzed on a 1% agarose gel in 1× TAE at 7 V/cm for 30 minutes.

## Supporting Information

Methods S1 A detailed description of method employed for multiple sequence alignment, calculation of dissociation constant and estimation of secondary structure of protein.
(0.05 MB DOC)

Figure S1 A) Sequence alignment of HU protein from *M. tuberculosis* (M._tb_HU), *E. coli* (E._coli), *S. enterica* (S._enterica_Hup) and *B. subtilis* (B._subtilis_HU) using Clustal W programme shows conservation of N terminal region. Completely conserved residues are shaded green, identical residues are shaded yellow, similar residues are shaded cyan and different residues are white. B) Sequence alignment between *M. tuberculosis* HupB (M._tb_HU) and sea urchin Histone H1 (Sea_Urchin_H1-g) revealed conservation of amino acids at C terminal region. The rectangular shaded boxes represent conserved PAKK and KAAK amino acid residues.
(0.66 MB PDF)

Figure S2 Amino acid sequence alignment of HupB*_Mtb_* from *M. tuberculosis* (*M.tb*) with *M. bovis*, *M. bovis BCG*, *M. ulcerans*, *M. marinum*, *M. leprae*, *M. kansasii*, *M. smegmatis*, *M. avium*, *M. parascrofula*, *M. gilvium*, *M. vanbaalenii* and *M. abscessus*. Completely conserved residues are shaded green, identical residues are shaded yellow, similar residues are shaded cyan and different residues are white.
(1.35 MB PDF)

Figure S3 Plots indicating the half maximal saturation of A) 2- nucleotide gap DNA (high salt), B) 2- nucleotide gap DNA (low salt), C) Cruciform DNA (high salt), D) 1- nucleotide gap DNA (low salt), E) nick DNA (high salt), in presence of indicated HupB*_Mtb_*. Respective Kd values are shown as inset in each panel.
(1.89 MB EPS)

Figure S4 Plots indicating the half maximal saturation of A) nick DNA B) 1- nucleotide gap DNA, C) 2- nucleotide gap DNA, D) Cruciform DNA, in presence of indicated HupB*_MtbN_* in low salt conditions. Respective Kd values are shown as inset in each panel.
(1.54 MB EPS)

Figure S5 Far UV CD spectrum analysis of HupB*_Mtb_* and HupB*_MtbN_* was carried out as described in supplementary methods and shown by arrowheads. HupB*_Mtb_* has 8.3% as α helix, 50.8% as β sheet, 7.6% as turn and 33.3% as random, however HupB*_MtbN_* has 16.9% as α helix, 35.2% as β sheet, 16.9% as turn and 31.1% as random.
(1.36 MB EPS)
