# Supplementary material for: DNA Clasping by Mycobacterial HU: The C-Terminal Region of HupB Mediates Increased Specificity of DNA Binding
Source: PLoS One. 2010 Sep 2;5(9):e12551. doi: 10.1371/journal.pone.0012551 (PMC2932737; doi:10.1371/journal.pone.0012551)
Supplement: Methods S1 — A detailed description of method employed for multiple sequence alignment, calculation of dissociation constant and estimation of secondary structure of protein. (0.05 MB DOC) [file pone.0012551.s001.doc]

**Supplementary Methods 1**

**Sequence alignments**

All the sequences used in the manuscript were obtained from NCBI. Their individual NCBI reference numbers are [YP_979091.1](http://www.ncbi.nlm.nih.gov/sites/entrez?cmd=Retrieve&db=Protein&list_uids=121638867&dopt=GenPept&RID=26YBEHSP012&log$=prottop&blast_rank=4) for *M. bovis BCG*, [CAB46493.1](http://www.ncbi.nlm.nih.gov/sites/entrez?cmd=Retrieve&db=Protein&list_uids=5419919&dopt=GenPept&RID=26YBEHSP012&log$=prottop&blast_rank=5) for *M. bovis*, [NP_217502.1](http://www.ncbi.nlm.nih.gov/sites/entrez?cmd=Retrieve&db=Protein&list_uids=15610123&dopt=GenPept&RID=26YBEHSP012&log$=prottop&blast_rank=1) for *M. tuberculosis H37Rv*, [AAX89121.1](http://www.ncbi.nlm.nih.gov/sites/entrez?cmd=Retrieve&db=Protein&list_uids=62632033&dopt=GenPept&RID=26YBEHSP012&log$=prottop&blast_rank=6) for *M. ulcerans*, [YP_001850032.1](http://www.ncbi.nlm.nih.gov/sites/entrez?cmd=Retrieve&db=Protein&list_uids=183981741&dopt=GenPept&RID=26YBEHSP012&log$=prottop&blast_rank=8) for *M. marinum*, [NP_302157.1](http://www.ncbi.nlm.nih.gov/sites/entrez?cmd=Retrieve&db=Protein&list_uids=15827894&dopt=GenPept&RID=26YBEHSP012&log$=prottop&blast_rank=15) for *M. leprae*, [P_04747612.1](http://www.ncbi.nlm.nih.gov/sites/entrez?cmd=Retrieve&db=protein&dopt=GenPept&RID=26YBEHSP012&log$=protalign&blast_rank=13&list_uids=240168953) for *M. kansasii*, [YP_886729.1](http://www.ncbi.nlm.nih.gov/sites/entrez?cmd=Retrieve&db=Protein&list_uids=118471339&dopt=GenPept&RID=26YBEHSP012&log$=prottop&blast_rank=21) for *M. smegmatis*, [YP_883003.1](http://www.ncbi.nlm.nih.gov/sites/entrez?cmd=Retrieve&db=Protein&list_uids=118465605&dopt=GenPept&RID=26YBEHSP012&log$=prottop&blast_rank=18) for *M. avium*, [ZP_06852690.1](http://www.ncbi.nlm.nih.gov/sites/entrez?cmd=Retrieve&db=Protein&list_uids=296171286&dopt=GenPept&RID=26YBEHSP012&log$=prottop&blast_rank=16) for *M. parascrofulaceum*, [YP_001135482.1](http://www.ncbi.nlm.nih.gov/sites/entrez?cmd=Retrieve&db=Protein&list_uids=145224804&dopt=GenPept&RID=26YBEHSP012&log$=prottop&blast_rank=19) for *M. gilvum*, [YP_952958.1](http://www.ncbi.nlm.nih.gov/sites/entrez?cmd=Retrieve&db=Protein&list_uids=120403129&dopt=GenPept&RID=26YBEHSP012&log$=prottop&blast_rank=17) for *M. vanbaalenii*, [YP_001704022.1](http://www.ncbi.nlm.nih.gov/sites/entrez?cmd=Retrieve&db=Protein&list_uids=169630373&dopt=GenPept&RID=26YBEHSP012&log$=prottop&blast_rank=23) for *M. abscessus*, [NP_290632](http://www.ncbi.nlm.nih.gov/protein/15804591?report=genbank&log$=prottop&blast_rank=1&RID=270WWX9V012) for *E. coli* HupA, [NP_286182.1](http://www.ncbi.nlm.nih.gov/protein/15800170?report=genbank&log$=prottop&blast_rank=1&RID=2718GMAJ01S) for *E. coli* HupB, [NP_390160.1](http://www.ncbi.nlm.nih.gov/protein/16079336?report=genbank&log$=prottop&blast_rank=1&RID=271GA2V7012) for *B. subtilis* HU and [NP_999720.1](http://www.ncbi.nlm.nih.gov/protein/NP_999720.1) for sea urchin (*Strongylocentrotus purpuratus*) histone H1. ClustalW software carried out the multiple sequence alignments.

**Calculation of dissociation constant**

Kinetics of dissociation was calculated by using probe 1 nM and protein concentration in the range as indicated in the respective figures. The image analysis was carried out using Image Quant software. The percentage of bound probe was plotted against protein concentration. The percentage of bound probe was calculated as follows:

(% of bound probe) = 100 - (% of free probe)

The graph of bound probe versus protein concentration was plotted using Sigma plot software version 10. Curve fitting was carried out by utilizing least square hypothesis [1]

**Determination of Secondary Structure of HupB*_Mtb_* and HupB*_MtbN_* by Far-UV Circular Dichroism Spectroscopy**

Far-UV CD spectra were recorded at 25 ^0^C in a cuvette of 0.1 cm path length and at 2nm bandwidth using a JASCO J-810 spectrophotometer. All spectra were recorded from 200 to 250 nm wavelength range, having a response time of 2 sec with a scan speed of 100 nM min^-1^. Spectra were signal averaged by adding at least three accumulations [2,3]. Subtracting spectra of respective buffer blanks, obtained under identical conditions, made a baseline correction. Percentage of secondary structure was calculated, using spectra max software of JASCO.

**References**

1. Pinson V, Takahashi M, Rouviere-Yaniv J (1999) Differential binding of the *Escherichia coli* HU, homodimeric forms and heterodimeric form to linear, gapped and cruciform DNA. J Mol Biol 287: 485-497.

2. Tundup S, Akhter Y, Thiagarajan D, Hasnain SE (2006) Clusters of PE and PPE genes of *Mycobacterium tuberculosis* are organized in operons: evidence that PE *Rv2431c* is co-transcribed with PPE *Rv2430c* and their gene products interact with each other. FEBS Lett 580: 1285 - 1293.

3. Choudhary RK, Pullakhandam R, Ehtesham NZ, Hasnain SE (2004) Expression and characterization of *Rv2430c*, a novel immunodominant antigen of *Mycobacterium tuberculosis*. Protein Expr Purif 36: 249 –253.
