# Supplementary material for: DNA Clasping by Mycobacterial HU: The C-Terminal Region of HupB Mediates Increased Specificity of DNA Binding
Source: PLoS One. 2010 Sep 2;5(9):e12551. doi: 10.1371/journal.pone.0012551 (PMC2932737; doi:10.1371/journal.pone.0012551)
Supplement: Figure S1 — A) Sequence alignment of HU protein from M. tuberculosis (M._tb_HU), E. coli (E._coli), S. enterica (S._enterica_Hup) and B. subtilis (B._subtilis_HU) using Clustal W programme shows conservation of N terminal region. Completely conserved residues are shaded green, identical residues are shaded yellow, similar residues are shaded cyan and different residues are white. B) Sequence alignment between M. tuberculosis HupB (M._tb_HU) and sea urchin Histone H1 (Sea_Urchin_H1-g) revealed conservation of amino acids at C terminal region. The rectangular shaded boxes represent conserved PAKK and KAAK amino acid residues. (0.66 MB PDF) [file pone.0012551.s002.pdf]

Supplementary Figure 1

A

```
                  1                                                          60
E._coli_HupB      MNKSQLIDKIAAGADISKAAAGRALDAIIASVTESLKEGDDVALVGFGTFAVKERAARTG
S._enterica_Hup   MNKSQLIEKIAAGADISKAAAGRALDAIIASVTESLKEGDDVALVGFGTFAVKERAARTG
E._coli_HupA      MNKTQLIDVIAEKAELSKTQAKAALESTLAAITESLKEGDAVQLVGFGTFKVNHRAERTG
B._subtilis_HU    MNKTELINAVAEASELSKKDATKAVDSVFDTILDALKNGDKIQLIGFGNFEVRERSARKG
M._tb_HU          MNKAELIDVLTQKLGSDRRQATAAVENVVDTIVRAVHKGDSVTITGFGVFEQRRRAARVA
consensus         MNKsqLId-ia-gadisk--A-rAld-iiasiteslkeGD-v-lvGFGtF-vkeRaaRtg

                  61                           90
E._coli_HupB      RNPQTGKEITIAAAKVPSFRAGKALKDAVN------------------------------
S._enterica_Hup   RNPQTGKEITIAAAKVPSFRAGKALKDAV-------------------------------
E._coli_HupA      RNPQTGKEIKIAAANVPAFVSGKALKDAVK------------------------------
B._subtilis_HU    RNPQTGEEIEIPASKVPAFKPGKALKDAVAGK----------------------------
M._tb_HU          RNPRTGETVKVKPTSVPAFRPGAQFKAVVSGAQRLPAEGPAVKRGVGASAAKKVAKKAPA
consensus         RNPqTGkei-iaaakVPaFr-GkalKdaV-------------------------------

E._coli_HupB      ------------------------------------------------------------
S._enterica_Hup   ------------------------------------------------------------
E._coli_HupA      ------------------------------------------------------------
B._subtilis_HU    ------------------------------------------------------------
M._tb_HU          KKATKAAKKAATKAPARKAATKAPAKKAATKAPAKKAVKATKSPAKKVTKAVKKTAVKAS
consensus         ------------------------------------------------------------

E._coli_HupB      ----------------------------------
S._enterica_Hup   ----------------------------------
E._coli_HupA      ----------------------------------
B._subtilis_HU    ----------------------------------
M._tb_HU          VRKAATKAPAKKAAAKRPATKAPAKKATARRGRK
consensus         ----------------------------------
```

B

```
Sea_Urchin_H1-g   MSAAKPKVAKKARVAPAHPPSSQMVVAAVTALKERGGSSTQAIKKYIAANYTVDMTKQGP
M._tb_HU          MNKAELIDVLTQKLGSDRRQATAAVENVVDTIVR----AVHKGDSVTITGFGVFEQRRR-
consensus         M--A--------rva------s--V---V--l--rggs------------y-V---k--p

Sea_Urchin_H1-g   FIRRALVKGVASGALVQTKGKGASGSFKLGKKKEGKSDAQKARIAAKKAKLAAKKKEQRE
M._tb_HU          --AARVARNPRTGETVKVKPTSVP-AFRPGAQFKAVVSGAQRLPAEGPAVKRGVGASAAK
consensus         fi---l-k---sG--V--K-----g-Fk-G----g---a-----A---A---a-------

Sea_Urchin_H1-g   KKALKTKARKEKVAAKKAAKKATKKTKKVKKPAAKKAKKPAAKKP-AAKKPAAKKAKKPA
M._tb_HU          KVAKKAPAKKATKAAKKAATKAPARKAATKAPAKKAATKAPAKKAVKATKSPAKKVTKAV
consensus         K-A-K--ArK---AAKKAA-KA--k----K-PA-K-A-K--AKK-v-A-K--AKK--K--

Sea_Urchin_H1-g   KKVAKPA---KKAAAKPAKKAAKPAKKAAKPAKKAAKPAKK-
M._tb_HU          KKTAVKASVRKAATKAPAKKAAAKRPATKAPAKKATARRGRK
consensus         KK-A--AsvrK-A---PAKKAA--------PAKKA-----kk
```
