# Supplementary material for: DNA Clasping by Mycobacterial HU: The C-Terminal Region of HupB Mediates Increased Specificity of DNA Binding
Source: PLoS One. 2010 Sep 2;5(9):e12551. doi: 10.1371/journal.pone.0012551 (PMC2932737; doi:10.1371/journal.pone.0012551)
Supplement: Figure S2 — Amino acid sequence alignment of HupBMtb from M. tuberculosis (M.tb) with M. bovis, M. bovis BCG, M. ulcerans, M. marinum, M. leprae, M. kansasii, M. smegmatis, M. avium, M. parascrofula, M. gilvium, M. vanbaalenii and M. abscessus. Completely conserved residues are shaded green, identical residues are shaded yellow, similar residues are shaded cyan and different residues are white. (1.35 MB PDF) [file pone.0012551.s003.pdf]

Supplementary Figure 2

```
M._bovis_BCG_HU -----MNKAELIDVLTQKLGSDRRQATAAVENVVDTIVRAVHKGDSVTITGFGVFEQRRR
M._bovis_HU     -----MNKAELIDVLTQKLGSDRRQATAAVENVVDTIVRAVHKGDSVTITGFGVFEQRRR
M._tb_HU        -----MNKAELIDVLTQKLGSDRRQATAAVENVVDTIVRAVHKGDSVTITGFGVFEQRRR
M._ulcerans_HU  -----MNKAELIDVLTQKLGSDRRQATAAVENVVDTIVRAVHKGDSVTITGFGVFEQRRR
M._marinum_HU   -----MNKAELIDVLTQKLGSDRRQATAAVENVVDTIVRAVHKGDSVTITGFGVFEQRRR
M._leprae_HU    -----MNKAELIDVLTQKLGSDRRQATAAVENVVDTIVRAVHKGDSVTITGFGVFEQRRR
M._kansasii_HU  -----MNKAELIDVLTQKLGSDRRQATAAVENVVDTIVRAVHKGDSVTITGFGVFEQRRR
M._smegmatis_HU -----MNKAELIDVLTTKMGTDRRQATAAVENVVDTIVRAVHKGDSVTITGFGVFEQRRR
M._avium_HU     MSEGLMNKAELIDVLTQKLNTDRRQATAAVENVVDTIVRAVHKGDSVTITGFGVFEQRRR
M._parascrofula -----MNKAELIDVLTQKLNTDRRQATAAVENVVDTIVRAVHKGDSVTITGFGVFEQRRR
M._gilvum_HU    -----MNKAELIDVLTEKLGSDRRQATAAVENVVDTIVRAVHKGESVTITGFGVFEQRRR
M._vanbaalenii_ -----MNKAELIDVLTEKLGSDRRQATAAVENVVDTIVRAVHKGESVTITGFGVFEQRRR
M._abscessus_HU -----MNKAELIDVLTQKLGSDRRQATAAVEHVVDTIVRTVHKGESVTITGFGVFEQRRR
consensus       -----MNKAELIDVLTqKlgsDRRQATAAVEnVVDTIVRaVHKGdSVTITGFGVFEQRRR

M._bovis_BCG_HU AARVARNPRTGETVKVKPTSVPAFRPGAQFKAVVSGAQRLPAEGPAVKRGVGAS-AAKKV
M._bovis_HU     AARVARNPRTGETVKVKPTSVPAFRPGAQFKAVVSGAQRLPAEGPAVKRGVGAS-AAKKV
M._tb_HU        AARVARNPRTGETVKVKPTSVPAFRPGAQFKAVVSGAQRLPAEGPAVKRGVGAS-AAKKV
M._ulcerans_HU  AARVARNPRTGETVKVKPTSVPAFRPGAQFKAVVSGAQRLPAEGPAVKRGVMASAAAKKA
M._marinum_HU   AARVARNPRTGETVKVKPTSVPAFRPGAQFKAVVSGAQRLPAEGPAVKRGVMASAAAKKA
M._leprae_HU    AARVARNPRTGETVKVKPTSVPAFRPGAQFKAVVAGAQRLPLEGPAVKRGVATS-AAKKA
M._kansasii_HU  AARVARNPRTGETVKVKPTSVPAFRPGAQFKAVVAGAQKLPAEGPAVKRGVGTS-AAKKA
M._smegmatis_HU AARVARNPRTGETVKVKPTSVPAFRPGAQFKAVISGAQKLPADGPAVKRGVTA-GPAKK-
M._avium_HU     AARVARNPRTGETVKVKPTSVPAFRPGAQFKAVVSGAQRLPSEGPAVKRGVVG-GAAKKT
M._parascrofula AARVARNPRTGETVKVKPTSVPAFRPGAQFKAVVSGAQRLPSEGPAVKRGVVASGAAKKT
M._gilvum_HU    AARVARNPRTGETVKVKPTSVPAFRPGAQFKAVVSGAQKLPAEGPAVKRGVAAASTARKA
M._vanbaalenii_ AARVARNPRTGETVKVKPTSVPAFRPGAQFKAVVSGAQKLPAEGPAVKRGVTATSTARKA
M._abscessus_HU AARVARNPRTGETVKVKPTSVPTFRPGAQFKAVVSGAQKLPADGPAVKRGSTAAPAKRAA
consensus       AARVARNPRTGETVKVKPTSVPaFRPGAQFKAVvsGAQrLPaeGPAVKRGv-as-aakka

M._bovis_BCG_HU AKK---------APAKKAT---KAAKKAATK-----APA---------KKAAT-KA----
M._bovis_HU     AKK---------APAKKAT---KAAKKAATK-----APA---------KKAAT-KA----
M._tb_HU        AKK---------APAKKAT---KAAKKAATK-----APARKAATKAPAKKAAT-KA----
M._ulcerans_HU  AKK---------APAKKAAT-KTAAKKAATK-----APAKKAATKAPAKKAAT-KARAKK
M._marinum_HU   AKK---------APAKKAAT-KTAAKKAATK-----APAKKAATKAPAKKAAT-KA----
M._leprae_HU    AIK--------KAPVK-----KALAKKAATK-----APA----------KKAV-KA----
M._kansasii_HU  AKKAPARKAATKAPAKKAAT-KAPAKKAATK-----APA----------KTAA-KA----
M._smegmatis_HU AAK--------KAPAKKAAAKKT-ATKAAAK----KAPA---------KKAAT-KA----
M._avium_HU     AAK--------KAPAKKAAAKKAPAKKAAAK----KAPA---------KKAAVKKA----
M._parascrofula AAK--------KAPAKKAAAKKTAAKKAPAKKAATKAPA---------KKAAT-KA----
M._gilvum_HU    AKK---------APAKKAAP----AKKTAAKK---AAPA----------KKAATKA----
M._vanbaalenii_ AKK---------APAKKAA-----VKKAAPAK---KAPA----------KKAA-------
M._abscessus_HU AKK---------AAPAKKAP----AKKAAPAK---KAPV----------KKAV-------
consensus       AkK---------Apakkaa--k--akKaatk-----APa---------kKaAt-ka----

M._bovis_BCG_HU -----PAKKAV-KATKSPAKK-VTK--A-VKK---TAVKASVRKA-ATKAPAKKAAAK--
M._bovis_HU     -----PAKKAV-KATKSPAKK-VTK--A-VKK---TAVKASVRKA-ATKAPAKKAAAK--
M._tb_HU        -----PAKKAV-KATKSPAKK-VTK--A-VKK---TAVKASVRKA-ATKAPAKKAAAK--
M._ulcerans_HU  AATKAPAKKAATKVTKAPAKK-VTK--ATVKK---TAAKAPVRKG-ATKAPAKKAAAK--
M._marinum_HU   -----PAKKAVTKVTKAPAKK-VTK--ATVKK---TAAKAPVRKA-ATKAPAKKAAAK--
M._leprae_HU    -----PAKKITT-AVKVPAKK-ATK--V-VKK---VAAKAPVRKA-TTRALAKKAAVK--
M._kansasii_HU  -----PAKKAATKATKAPAKA-TKT--T-AKK---AAAKAPVRKA-ATKAPAKKAAAK--
M._smegmatis_HU -----PAKKAA---TKAPAKKAATK--APAKK---AATKAPAKKA-AAKAPAKKAATK--
M._avium_HU     -----PARKAA---TKAPVRKAATK--APAKKV--AAKKAPAKKA-ATKAPAKKAASK--
M._parascrofula -----PAKKAA---TKAPVKKAATK--APVKK-------AAAKKA-ATKAPVKKAAAK--
M._gilvum_HU    -----PAKKAAPAKKAAPAKKTAAKKAAPAKKAPAAKKAAPAKKAPAKKAATKAAPAKK-
M._vanbaalenii_ -----PAKKAA-VKKAAPAKKAPAKKAAPAKKA-AVKKAAPAKKAPAKKAAVKKAPAKKA
M._abscessus_HU ------VKKAAPVKK-APVKKAVVKKAAPVKKA---VTKAPAKKA-ATKAPAKKAATK--
consensus       -----pakKaa---tkaPakk--tk--a-vKK----a-kApvrKa-atkApaKkAaaK--
```
